# quercusTOA: integrating functional annotations and comparative genomics across oak lineages

**DOI:** 10.3389/fbinf.2026.1821531

**Published:** 2026-06-01

**Authors:** Fernando Mora-Márquez, Mikel Hurtado, Unai López de Heredia

**Affiliations:** 1 GI en Desarrollo de Especies y Comunidades Leñosas (WooSP), Dpto. Sistemas y Recursos Naturales, ETSI Montes, Forestal y del Medio Natural, Universidad Politécnica de Madrid, Madrid, Spain; 2 Dep. of Immunology and Cell Biology, Université de Sherbrooke, Sherbrooke, QC, Canada; 3 Dep. Biología Vegetal y Ecología. Facultad de Ciencia y Tecnología. Euskal Herriko Unibertsitatea (EHU), Leioa, Spain

**Keywords:** comparative genomics, forest genetics, functional annotation, genomic lift-over, orthology, *Quercus*, SQLite database

## Abstract

Oaks (*Quercus* L.) are key components of Northern Hemisphere Forest ecosystems, yet the integration of their rapidly growing genomic resources remains challenging. Here, we present quercusTOA, a genomic and functional resource that integrates nine *Quercus* genome assemblies. By combining automated functional annotation (InterProScan, eggNOG-mapper) with comparative genomics via genomic lift-over, we have developed a relational database designed to link protein-centric annotations with positional genomic data. Our results demonstrate that this integration supports cross-species ortholog identification and synteny analysis. To facilitate data access and exploration, we provide the quercusTOA-app, a user-friendly interface that streamlines database management and specific bioinformatic tasks. These include functional annotation, sequence-based homology searches, and multiple sequence alignments. Furthermore, the application enables the construction of phylogenetic trees for individual orthologous genes and proteins, allowing for the study of specific sequence evolution across the included assemblies. quercusTOA provides a standardized and scalable framework for evolutionary and functional studies in *Quercus*, offering a consistent approach to maintain genomic coordinate synchronization across the genus.

## 1 Introduction

Oaks (*Quercus* L., Fagaceae) are a woody angiosperm clade including about 400–500 species. They are among the most widespread and diverse tree genera in Northern Hemisphere Forest ecosystems, including temperate deciduous forests, subtropical and tropical savannas, cloud forests, and tropical montane or Mediterranean forests (Nixon, 2006). The economic significance of oaks stems from fruit exploitation for food and extensive livestock farming, as well as from the extraction of wood and other sustainable products like cork, for various uses. The infrageneric classification of the genus is a subject of controversy (Denk et al., 2017), partly due to the extraordinary hybridization ability of these allogamous species (Whittemore and Schaal, 1991). For all these reasons, oaks have garnered significant attention from botanists, evolutionary biologists, forests breeders, and biotechnologists with a special focus on molecular data in recent decades. Indeed, genome and transcriptome sequencing is shedding light on oak evolution and diversification (Larson et al., 2025), historical and contemporary hybridization (López de Heredia et al., 2017), and their response to stress (Escandón et al., 2021), among other aspects. Furthermore, from a more applied perspective, it has driven the development of molecular markers associated with traits of interest (Nagamitsu et al., 2024).

Genomic resources for oak species are continuously expanding due to the ongoing production of various research groups. Since the seminal *Quercus lobata* genome assembly draft became publicly available (Sork et al., 2016), numerous oak genome assemblies of diverse quality have been released (Zhang et al., 2025). While some of these genome assemblies are drafts, presenting only data at the scaffold level -e.g., *Q. suber* (Ramos et al., 2018)-, others have reached chromosome level and are classified as high-quality references. This progress is possible by the continuous development of sequencing technologies, such as HI-C conformation capture (Belton et al., 2012) and the combination of short and long read sequencing (Wang et al., 2023). Additionally, the optimization of assemblies, gene discovery, and annotation has been enhanced by advances in novel bioinformatic algorithms (Li and Durbin, 2024).

This impressive explosion of whole-genome sequencing studies in oaks has yielded a vast number of genomic resources. These are essential for advancing our understanding of oak genetics and epigenetics, not only to unravel the evolution of the genus but also to enable more applied research on specific genes or gene families. However, public access to sequence data and associated information is often limited. This is due to the scattered storage of genomic data across separate repositories. While some data is available through major public archives like the National Center for Biotechnology Information (NCBI) (Sayers et al., 2026), the European Nucleotide Archive (ENA) (Burgin et al., 2023), and the National Genomics Data Center of the China National Center for Bioinformation (CNCB-NGDC) (CNCB–NGDC Members and Partners, 2026), finding comprehensive resources can be challenging. Furthermore, specialized, single-species databases, such as CorkOakDB for *Q. suber* (Arias-Baldrich et al., 2020), and broader tree genomics resources like TreeGenes (Falk et al., 2018), which often contain genetic maps and comparative genomics data, also host valuable oak genomic information, along with various institutional or project-specific repositories.

Unfortunately, this fragmentation of genomic data across public repositories often leads to critical issues for the research community. Firstly, there is variable assembly quality, meaning the integrity and accuracy of available genome assemblies can differ significantly across platforms (Amarasinghe et al., 2020). Secondly, information redundancy is a frequent problem, where identical or highly similar data might be present in multiple, disparate locations, complicating data retrieval and increasing the risk of inconsistencies (Chen et al., 2017). Furthermore, outdated or incorrect annotations pose a significant challenge, as gene annotations might be incomplete, erroneous, or not regularly updated, hindering downstream analyses (Vuruputoor et al., 2023). For example, Larson et al. (2025) suggested that previously reported expansions and contractions of disease-resistance gene families in oaks might be artifacts of technical differences in repeat annotation and gene identification rather than true biological variation. Lastly, access is hampered by limited centralized resources; for instance, the Quercus Portal (Quercus Portal, 2024), while intended as a central hub, may suffer from outdated links or incomplete coverage of the most recent genomic resources. The absence of a standardized bioinformatic framework that synchronizes physical genomic coordinates with functional metadata remains a major technological barrier, as it prevents researchers from performing seamless synteny studies or large-scale evolutionary analyses of gene families. Moreover, only a subset of available assemblies provides publicly accessible gene annotation and protein files. This significantly restricts the utility of the associated genomic and functional information, thereby impeding collaborative research and comprehensive analyses within the broader scientific community.

In this manuscript, we introduce quercusTOA, an integrative framework designed to centralize and enhance access to the dispersed genomic resources of the genus *Quercus*. quercusTOA bridges existing data repositories by synchronizing protein-centric functional metadata with positional genomic data across nine genome assemblies from NCBI and CNCB-NGDC. Building upon the architectural principles of TOA-Taxonomy Oriented Annotation (Mora-Márquez et al., 2021a) and gymnoTOA (Mora-Márquez et al., 2025), this resource incorporates a taxonomy-aware approach that enables the classification of gene clusters based on their evolutionary conservation. Specifically, the database allows researchers to distinguish between ‘core orthologs’ shared across the genus and lineage-specific genes unique to particular infrageneric sections. Accompanied by the quercusTOA-app, a user-friendly interface for local data exploration, this system ensures scalability to accommodate the rapidly expanding volume of oak genomic resources.

## 2 Methods

### 2.1 Data acquisition

**FIGURE 1:**
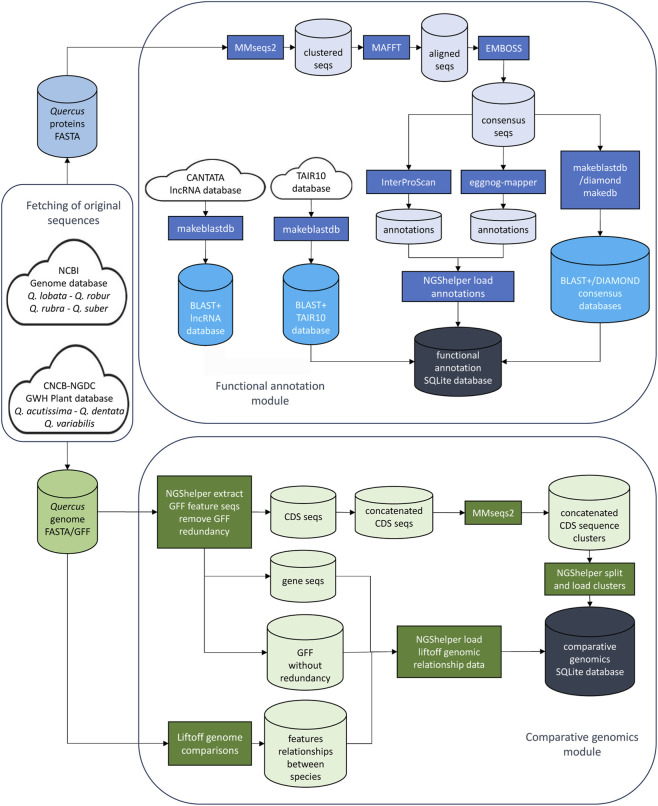
quercusTOA construction workflow. Schematic representation of the database pipeline. The functional annotation module includes processing of *Quercus* protein sequences through clustering (MMseqs2), consensus generation (EMBOSS), and functional assignment using InterProScan and eggNog-mapper against specialized databases. The comparative genomics module includes workflow for genome-wide analysis, including GFF normalization (NGShelper), gene mapping (Liftoff), and ortholog clustering (MMseqs2). Processed data are integrated into modular SQLite databases for downstream analysis.

quercusTOA was constructed by integrating data from the increasing number of *Quercus* genome assemblies available through the NCBI Genomes database and the CNCB-NGDC Plant Genome Warehouse repository. In the first stage of the pipeline, *Quercus* genome and protein FASTA sequences were fetched for species with complete, publicly available genome assembly records (Table 1). The most recent genome assemblies for *Q. suber* (Usié et al., 2023), *Q. robur* (Plomion et al., 2016), *Q. lobata* (Sork et al., 2016), and *Q. rubra* (Kapoor et al., 2023) were downloaded from the NCBI Genomes database. The retrieved data included the genome assemblies in FASTA format, their corresponding annotation GFF files, and the protein FASTA sequences. Similarly, equivalent data were downloaded from the CNCB-NGDC Plant Genome Warehouse for the assemblies of *Q. variabilis* (Liang et al., 2025), *Q. dentata* (Wang et al., 2023), *Q. gilva* (Zhou et al., 2022), *Q. longispica* (Ren et al., 2025) and *Q. acutissima* (Liu et al., 2022). The genome and protein sequences were then loaded into the sequences.db SQLite database file (Figure 1) using the load-species-seqs.py utility from the NGShelper suite (Mora-Márquez et al., 2021b).

**TABLE 1:** Species and genome assemblies integrated in QuercusTOA.

Species	Assembly version	Source	Data citation
*Quercus acutissima*	GWHBGBO00000000	CNCB-NGDC	CNCB–NGDC (2025a)
*Quercus dentata*	GWHBRAD00000000	CNCB-NGDC	CNCB–NGDC (2025b)
*Quercus gilva*	GWHDOCW00000000	CNCB-NGDC	CNCB–NGDC (2025c)
*Quercus longispica*	GWHESEV00000000	CNCB-NGDC	CNCB–NGDC (2025d)
*Quercus variabilis*	GWHEQCV00000000	CNCB-NGDC	CNCB–NGDC (2025e)
*Quercus lobata*	GCF_001633185.2_ValleyOak3.2	NCBI	NCBI Genome (2019)
*Quercus robur*	GCF_932294415.1_dhQueRobu3.1	NCBI	NCBI Genome (2022)
*Quercus rubra*	GCA_035136125.1_Qrubra_687_v2.0	NCBI	NCBI Genome (2024a)
*Quercus suber*	GCF_002906115.3_Cork_oak_2.0	NCBI	NCBI Genome (2024b)

List of the nine *Quercus* species included, specifying assembly version, accession numbers (NCBI/CNCB-NGDC), and specific dataset citation. Most genome assemblies are at the chromosome level, except for the *Quercus suber* assembly, which is at the scaffold level.

### 2.2 Genomic mapping and redundancy filtering

To take advantage of the information retrieved from genome repositories, the FASTA genome files and their corresponding GFFs included in the sequence dataset were then analyzed using Liftoff (Shumate and Salzberg, 2021) to set up Coding Sequences (CDS) and gene correspondence among the species. We relied on original methods of gene inference employed by the authors of the genome assemblies rather than using a single protocol to perform unified gene inference. However, the GFF file was previously processed by remove-gff-redundancies.py (NGShelper) to obtain a GFF without redundancies (mRNAs with the same concatenated CDS sequence and their corresponding features within a gene). This redundancy filtering was particularly critical for assemblies with high levels of alternative transcript predictions, such as *Q. robur* and *Q. lobata*, ensuring that the subsequent mapping and ortholog clustering were based on a single, representative protein per locus when identical sequences were present. Subsequently, we employed Liftoff to map Gene and Coding Sequence (CDS) coordinates across genomes. These correspondences were loaded into the comparative-genomics.db SQLite database using the get-liftoff-gff-data.py and load-liftoff-gff-data.py scripts (NGShelper). Separately, the extract-gff-feature-seqs.py script (NGShelper) was used to extract gene, CDS, and concatenated CDS, which were subsequently clustered using MMseqs2. The resulting cluster sequences were loaded into the database by the split-mmseqs2-concds-clusters.py and load-mmseqs2-concds-clusters.py scripts (NGShelper). Finally, genomic relationships and homologous proteins were retrieved using the get-liftoff-genome-relationships.py and get-liftoff-homologous-proteins.py scripts (NGShelper).

### 2.3 Functional annotation and multi-source integration

The protein sequences were concatenated and clustered using MMseqs2 ( Steinegger and Söding, 2017 ), Mafft (Katoh and Standley, 2013 ) and Emboss (Rice et al., 2000). This process yielded a ‘clustered consensus proteins’ file that was then used to obtain functional annotation features and to build Blast+ (Camacho et al., 2009) and Diamond (Buchfink et al., 2015) databases using makeblastdb and diamond makedb commands. Blast+ databases were also constructed to store homologs to the clustered sequences in *Arabidopsis* (TAIR10) (Berardini et al., 2015). These homologs were loaded into the functional-annotation.db SQLite database using the load-tair10-orthologs.py script (NGShelper). In addition, known plant long non-coding RNA (lncRNA) sequences for *Q. lobata*, *Q. suber*, and *A. thaliana* were sourced from CANTATA (Szcześniak et al., 2016) and processed to construct the ‘lncRNA’ Blast+ database. Finally, the database was populated with a comprehensive set of functional annotations and orthologous groups. These annotations, derived from InterProScan (Jones et al., 2014) and eggNOG-mapper (Cantalapiedra et al., 2021) searches, included: InterPro (Hunter et al., 2009), PANTHER (Thomas et al., 2003), and eggNOG (Hernández-Plaza et al., 2023) Gene Ontology (GO) terms; Enzyme Commission (E.C.) numbers; KEGG KOs, pathways, modules, classes, and reactions (Kanehisa et al., 2016); CAZy (Drula et al., 2022) and Pfam (Mistry et al., 2021) records; and MetaCyc metabolic pathway information (Caspi et al., 2014). Additionally, orthologous groups of proteins and genes corresponding to each consensus protein sequence were loaded. The overall quality of the consensus sequences was performed using BUSCO with the *embryophyta_odb10* gene set (Simão et al., 2015). Functional annotation features were loaded into the functional-annotation.db SQLite database using the load-interproscan-annotations.py and load-emapper-annotations.py scripts (NGShelper).

### 2.4 Computational infrastructure

To ensure reproducibility, the entire construction process was automated through a BASH script executed on an *AWS r5.8xlarge* instance. This script, along with the quercusTOA-app -a graphical interface developed to facilitate the exploration and analysis of the resulting resources (see Section 3.2)- are publicly available at the GitHub (https://github.com/GGFHF/quercusTOA-app) and Zenodo repositories (Mora-Márquez et al., 2026a). All SQLite databases were built and loaded using Python scripts from NGShelper.

### 2.5 Technical validation

#### 2.5.1 Sequence integrity and comparative genomics module

The quercusTOA approach enables a comprehensive comparison of the genomic space across the selected reference *Quercus* assemblies. To validate the architectural consistency of the processed data, we calculated basic statistics for each species, including the total number of genes, protein-coding genes, and the percentage of redundant mRNA identified during GFF analysis. Furthermore, we assessed the efficiency of the gene mapping process by calculating the mean percentage of genes successfully mapped with Liftoff after GFF correction. These metrics will provide a baseline for the comparative analysis of gene relationships and transcript redundancies across the genus. Technical validation of the comparative genomics module was performed by assessing the consistency of gene mapping and the identification of redundant mRNA sequences across nine *Quercus* species.

In addition, to evaluate the genomic consistency and conservation of quercusTOA across the nine *Quercus* species, we performed a comparative distribution analysis of shared gene clusters and orthogroups (OGs) to detect core orthologs and species-specific OGs and gene clusters using five distinct methodologies: First, “oak gene clusters” were defined based on the homology relationships between the nine genomes. Functional orthology was assessed by mapping *Quercus* protein clusters to the eggNOG and TAIR databases within the quercusTOA framework. We identified orthologs based on homology with the *A. thaliana* proteome (TAIR). Additionally, protein clusters were assigned to eggNOG OGs at two hierarchical taxonomic levels: Streptophyta (representing broad plant conservation) and order (representing lineage-specific conservation). Finally, we used the alignment-based *de novo* approach of OrthoFinder (Emms and Kelly, 2019) on the original protein files of each genome. To minimize redundancy and ensure computational efficiency, the input protein sets were filtered to include only the longest isoform per gene for each species. For each method, we calculated the frequency of gene clusters/OGs shared by a specific number of species, ranging from 1 (species-specific) to 9 (core genome). The resulting distribution was expressed as the percentage of the total gene clusters/OGs identified by each method to allow for direct comparison of genomic stability and database curation quality.

#### 2.5.2 Functional annotation and benchmarking

We evaluated two database groups: DB1, generated using a protein-centric protocol analogous to gymnoTOA-DB (Mora-Márquez et al., 2025), and DB2, constructed using annotated genome assemblies from NCBI and CNCB-NGDC repositories. While DB1 relies on a broad retrieval of *Quercus* protein sequences (taxid:3511) from multiple sources -including UniProt/SwissProt (The Uniprot Consortium, 2017), PIR (George et al., 1997), PRF (Protein Research Foundation, 2026), PDB (Burley et al., 2025), GenBank (Sayers et al., 2025), and RefSeq (O’Leary et al., 2016)- DB2 integrates protein/GFF data from genome assemblies to enable advanced comparative genomics features.

To determine the optimal configuration, we benchmarked five combinations of MMseqs2 coverage (-c) and identity (--min-seq-id) thresholds for both groups. Sensitivity and completeness were assessed by comparing total consensus sequences, functional assignments (InterProScan and eggNOG-mapper), *A. thaliana* (TAIR10) homology, and BUSCO scores.

#### 2.5.3 Functional recovery in non-model oak species

To evaluate the practical utility of quercusTOA for species lacking a reference genome, we simulated a “degenerated” transcript set using 111 EST sequences from *Q. pubescens* available in Treegenes (Treegenes Database, 2026). We employed the debase-transcript-sequences.py script from the NGShelper suite to introduce realistic biological and sequencing noise, including fragmentation (*prob* = 0.3), single-nucleotide mutations (*prob* = 0.5), and indels (*prob* = 0.25). The resulting sequences were annotated using the quercusTOA-app on the quercusTOA-DB2-a database and cross-referenced with Trapid2.0 (Van Bel et al., 2013) (using *PLAZA Dicots 4.5*) (Van Bel et al., 2018) to benchmark performance against a standard plant genomics platform.

A critical challenge in benchmarking these pipelines is the lack of standardized nomenclature across protein domain databases. While Trapid provides assignments primarily based on InterPro (IPR) accessions, quercusTOA integrates signatures from Pfam, eggNOG, and PANTHER. To ensure a fair comparison, Gene Ontology (GO) terms were adopted as the universal functional language for validation.

## 3 Results

### 3.1 Database architecture and content

The quercusTOA database is hosted at the UPMdrive cloud repository (Universidad Politécnica de Madrid, 2014–2026) and is permanently archived in the Zenodo repository (Mora-Márquez et al., 2026b). The data can be accessed and downloaded via the project website (https://blogs.upm.es/quercustoa/) or using the quercusTOA-APP software available at the GitHub (https://github.com/GGFHF/quercusTOA-app) and Zenodo repositories (Mora-Márquez et al., 2026a). The dataset consists of three relational SQLite databases (sequences.db, functional-annotation.db, and comparative-genomics.db), two Blast+ databases (‘lncRNA’, and ‘clustered consensus oak proteins’), and one Diamond database (‘clustered consensus oak proteins’) (Table 2). To facilitate data integration, the three SQLite databases share a common relational structure where the protein identification acts as the unique connector across all datasets (Supplementary Material 1). While this modular architecture allows for advanced SQL querying, the quercusTOA-app provides a user-friendly interface to exploit these relationships. Beyond simple data retrieval, the application allows users to input protein or transcript sequences to identify homologous genes and proteins across the nine *Quercus* species, automatically performing multiple sequence alignments of the recovered orthologs, and retrieving functional data. This functionality effectively bridges the gap between raw genomic data and comparative evolutionary analysis.

**TABLE 2:** Summary of data records and database architecture within QuercusTOA.

Data record	File name	Format	Description
Raw sequences	sequences.db	SQLite	Contains raw protein and gene sequences for available *Quercus* species
Functional annotation	functional-annotation.db	SQLite	Includes protein clusters, GO terms, metabolic pathways, and *A. thaliana* orthologs
Comparative genomics	comparative-genomics.db	SQLite	Stores gene/CDS correspondences, homologous proteins, and unmapped genes
lncRNA BLAST+ DB	lncRNA	Blast+	Database for long non-coding RNA sequences
Clustered proteins	clustered_consensus_oak_proteins	Blast+/Diamond	Consensus protein sequences for clustered oak orthologs

The database integrates structured genomic and proteomic resources for *Quercus* species across three primary SQLite modules: sequences.db, containing raw protein and gene sequences; functional-annotation.db, which includes protein clusters, GO terms, metabolic pathways, and *Arabidopsis thaliana* orthologs; and comparative-genomics.db, storing gene/CDS correspondences and homologous protein relationships. Additionally, the repository provides specialized Blast+ and Diamond databases for long non-coding RNA (lncRNA) sequences and consensus protein sequences derived from clustered oak orthologs.

**TABLE 3:** Summary of genomic features and Liftoff mapping statistics for the nine *Quercus* species.

Species	#Genes	#Proteins in coding genes	Redundant mRNA (n)	Redundant mRNA (%)	Mean% targeted maps liftoff (s.d.)	Mean% reference maps liftoff (s.d.)
*Quercus acutissima*	31,490	31,040	0	0.00	86.91 (3.62)	92.81 (1.10)
*Quercus dentata*	28,626	35,836	2,906	8.11	90.32 (2.55)	88.14 (1.18)
*Quercus gilva*	40,166	40,166	0	0.00	88.93 (2.94)	92.18 (0.87)
*Quercus lobata*	41,698	53,226	8,126	15.27	89.58 (2.72)	87.32 (1.87)
*Quercus longispica*	35,901	35,901	0	0.00	90.12 (3.18)	84.79 (1.10)
*Quercus robur*	41,859	53,760	10,303	19.16	89.59 (2.10)	84.54 (1.84)
*Quercus rubra*	33,247	47,694	6,205	13.01	87.84 (3.07)	89.20 (1.24)
*Quercus suber*	39,985	44,993	6,179	13.73	88.02 (3.91)	88.91 (0.77)
*Quercus variabilis*	34,681	34,681	0	0.00	89.38 (4.77)	92.80 (0.95)

The table summarizes the total gene counts, protein-coding genes, and total proteins within the GFF for each assembly. “Redundant mRNA” (n and %) refers specifically to mRNA, features within a single gene that encode for identical protein sequences, which were identified and normalized during our GFF analysis to ensure a non-redundant dataset. Mapping efficiency is presented via two metrics: “Mean targeted maps” represents the average percentage of genes successfully mapped to the species when it acts as the target for the other eight species. “Mean reference maps” represents the average success rate of the species’ own genes when mapped onto the other eight target species. Standard deviations (SD) are provided in parentheses.

The SQLite databases are organized into functional modules as follows:

**Sequence Database** (sequences.db)

This database serves as the repository for all raw sequence data:

- species_protein_seqs: Stores the full amino acid sequences for each protein ID and species.
- species_gene_seqs: Stores the nucleotide sequences for the identified genes.

**Comparative Genomics Database** (comparative-genomics.db)

This record stores the results of the genomic lift-over and comparative analysis among species:

- liftoff_gff_cds_data and liftoff_gff_gene_data: Detail the genomic coordinates (start, end, strand) and correspondences for CDS and genes between reference and target species.
- liftoff_homologous_proteins: Maps protein identifications between reference and target species.
- liftoff_unmapped_genes: Records genes from the reference species that could not be mapped to the target genome.
- mmseqs2_concatenated_cds_clusters: Links concatenated CDS sequences to their respective clusters, genes, and protein IDs.

**Functional Annotation Database** (functional-annotation.db)

This database contains the core functional information for the protein clusters. It is structured into the following tables:

- mmseq2_protein_clusters: Maps protein sequences to their respective cluster IDs and species.
- interproscan_annotations: Stores GO terms and metabolic pathway IDs derived from InterPro, PANTHER, MetaCyc, and Reactome.
- emapper_annotations: A comprehensive table including eggNOG orthologous groups, COG categories, KEGG information (KOs, pathways, modules, reactions), CAZymes, and Pfam families.
- tair10_info and tair10_orthologs: Contain *A. thaliana* peptide descriptions and their corresponding homology relationships with the oak protein clusters.
- go_ontology: A reference table for Gene Ontology term names and namespaces (biological process, molecular function, or cellular component).

### 3.2 The quercusTOA-app: a graphical interface for data exploration

To facilitate the exploration and analysis of the integrated resources, we developed the quercusTOA-app. Built in Python 3, this application features a cross-platform graphical user interface (GUI) compatible with Linux, Windows, and macOS, while also providing BASH scripts for high-throughput analysis on Linux servers. The application inherits robust functional annotation and enrichment utilities from the gymnoTOA-app framework (Mora-Márquez et al., 2025), including integrated BLAST/DIAMOND search engines and functional enrichment modules.

To ensure accessibility for users with limited bioinformatics expertise, quercusTOA-app automates the installation of required third-party dependencies and manages the direct download of the latest database versions from remote repositories. A key enhancement in this iteration is the Comparative Genomics Module, which enables homology searches via protein ID or sequence to retrieve consensus proteins alongside original sequences from all nine oak species. The tool automatically generates gene/protein FASTA files, multiple sequence alignments, and phylogenetic trees via Mafft. The application also allows users to supply their own genome assemblies to map annotations -using any database species as a reference through Liftoff- producing outputs that are fully compatible with LiftoffTools (Shumate and Salzberg, 2024) for further genomic analysis. Furthermore, quercusTOA-app can also run on Linux servers using the BASH scripts included in the software.

### 3.3 Technical validation

#### 3.3.1 Sequence integrity and comparative genomics module

The high success rate of gene mapping via Liftoff, with mean targeted map percentages consistently exceeding 86% (SD < 4.8) and mean reference maps above 84% (SD < 1.9), demonstrates the robustness of the cross-species annotation transfer (Table 3). Notably, the reference map percentages reached their highest values (>92%) in *Q. acutissima*, *Q. gilva*, and *Q. variabilis*, indicating that these chromosome-level assemblies provide highly reliable source annotations for the genus. Additionally, the quantification of redundant mRNAs -specifically defined as multiple mRNA features within a gene encoding identical protein sequences-highlights the effectiveness of our GFF analysis in normalizing disparate assembly formats. By identifying and removing 10,303 such redundant sequences in *Q. robur* and 8,126 in *Q. lobata*, the pipeline ensures that comparative metrics are not biased by identical duplicate entries in the original datasets.

**FIGURE 2:**
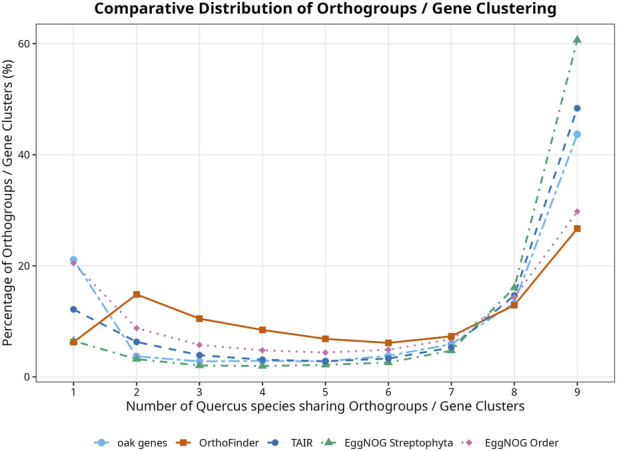
Comparative distribution of shared orthogroups and gene clusters across nine *Quercus* species. The plot illustrates the percentage of orthogroups/gene clusters (Y-axis) shared by an increasing number of species (X-axis), ranging from species-specific (1) to core orthogroups (9). Five different methodologies are compared: oak gene clusters, EggNOG OGs (OrthoGroups) at two taxonomic levels (Streptophyta and Order), TAIR (Arabidopsis-based orthology), and OrthoFinder orthogroups (*de novo* clustering).

The comparative analysis of the nine *Quercus* genomes included in quercusTOA revealed a characteristic U-shaped distribution in the sharing of orthogroups and gene clusters across all five methodologies. This pattern suggests a genomic architecture composed of a substantial conserved core alongside a fraction of lineage-specific or low-frequency clusters (Figure 2). The proportion of core orthogroups (n = 9) varied depending on the taxonomic scale and clustering stringency of each method. The highest conservation was identified at the eggNOG Streptophyta level (60.66%), followed by TAIR-based mapping (48.39%) and the oak gene clusters (43.70%). In contrast, the more lineage-specific eggNOG order level and the *de novo* OrthoFinder approach identified 29.77% and 26.71% of the clusters as core components, respectively. Regarding low-frequency clusters, the combined percentage of gene clusters/OGs present in only one or two species remained within a similar range across several independent methods: 21.12% for OrthoFinder, 24.80% for oak gene clustering, and 29.26% for eggNOG order. While the internal distribution between these two categories varied, with OrthoFinder showing a higher frequency at n = 2 (14.86%) compared to n = 1 (6.26%), and oak genes peaking at n = 1 (21.10%), the aggregate values for the accessory component were consistent across methodologies.

#### 3.3.2 Functional annotation and benchmarking

Our results demonstrate that DB2 achieves an annotation quality equivalent to DB1 while significantly expanding the sequence space (Table 4). The DB2-a parameter combination (MMseqs2 -c 1.0, --min-seq-id 1.000) was identified as the most comprehensive resource. While both configurations achieved high functional coverage, the DB2-a setup proved superior by enabling the direct synchronization of functional annotations with genomic coordinates through the Liftoff-derived mapping. This integration allows for a more comprehensive analysis than the protein-centric DB1 approach, as it facilitates the study of gene synteny and the identification of non-redundant genomic loci across the *Quercus* genus.

**TABLE 4:** Clustering and functional annotation statistics for *Quercus* genome assemblies.

Version	MMseqs2 (-c, --msi)	Original-seqs (n)	Clusters (n)	InterProScan	eggNOG-mapper	TAIR10	% w/o annot	BUSCO
DB1-a	1.0, 1.000	297,142	196,486	157,597 (3,967)	188,413 (7,410)	179,957 (18,999)	3.45%	1,614 (1,612)
DB1-b	1.0, 0.900	297,142	143,408	111,457 (3,949)	135,863 (7,373)	128,057 (18,837)	4.46%	1,614 (1,612)
DB1-c	0.0, 0.900	297,142	60,281	43,075 (3,406)	54,833 (7,011)	50,104 (16,671)	7.89%	1,614 (1,585)
DB1-d	0.9, 0.750	297,142	85,700	62,635 (3,855)	79,096 (7,214)	74,020 (18,166)	6.65%	1,614 (1,612)
DB1-e	0.9, 0.900	297,142	99,870	73,662 (3,895)	92,857 (7,316)	85,917 (18,421)	6.02%	1,614 (1,612)
DB2-a	1.0, 1.000	377,297	324,105	250,406 (4,711)	301,337 (7,584)	288,743 (20,038)	6.21%	1,614 (1,612)
DB2-b	1.0, 0.900	377,297	218,143	159,315 (4,697)	196,492 (7,539)	185,632 (19,834)	8.82%	1,614 (1,612)
DB2-c	0.0, 0.900	377,297	84,705	53,004 (3,645)	68,758 (7,036)	62,641 (17,049)	17.00%	1,614 (1,552)
DB2-d	0.9, 0.750	377,297	135,483	90,130 (4,595)	115,762 (7,401)	108,248 (19,254)	13.08%	1,614 (1,612)
DB2-e	0.9, 0.900	377,297	155,888	105,790 (4,646)	135,162 (7,480)	125,544 (19,477)	11.90%	1,614 (1,612)

The table summarizes the performance of the functional annotation module across different database versions and MMseqs2 clustering parameters (sequence identity and coverage). Statistics include the total number of input sequences and resulting non-redundant clusters, alongside functional assignment counts and Gene Ontology (GO) terms retrieved via InterProScan and eggNOG-mapper (unique records in parentheses). Homology results against the TAIR10 (*Arabidopsis thaliana*) reference (unique records in parentheses) and the percentage of unannotated clusters are provided. Assembly and annotation completeness are represented by BUSCO (*embryophyta_odb10*) scores, showing the total groups searched and the count of recovered complete genes (in parentheses).

Unlike clustered versions that prioritize size reduction, DB2-a preserves maximum functional diversity, identifying 7,584 clusters with distinct eggNOG-based assignments and 4,711 with unique InterProScan-based features. Remarkably, this functional breadth is highly comparable to the gold standard provided by the TAIR10 proteome, which identifies 7,734 and 5,709 clusters in the same categories, respectively.

A critical observation from the benchmarking process is the trade-off between database size reduction and functional information retention. As clustering parameters become more permissive -specifically in the DB2-c configuration where the coverage threshold is removed (-c 0.0)- the total number of clusters drops significantly to 84,705. However, this reduction in size is accompanied by a sharp increase in the percentage of unannotated clusters, which rises from 6.21% in DB2-a to 17.00% in DB2-c. This trend suggests that aggressive clustering leads to the loss of unique functional signatures.

Finally, DB2-a ensures full proteome representation, recovering 1,612 complete BUSCO genes (99.8% of the 1,614 searched). These metrics, combined with the identification of 288,743 clusters showing TAIR10 homology, reinforce the selection of DB2-a as the optimal balance for providing a high-resolution functional and comparative resource for *Quercus* genomics.

#### 3.3.3 Functional recovery in non-model oak species

**FIGURE 3:**
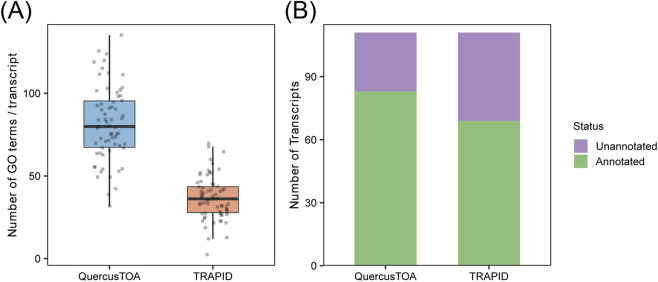
Benchmarking of annotation efficiency and functional richness. **(A)** Boxplot showing the distribution of GO term percentages by annotated transcripts. Despite the challenges in ontology mapping, quercusTOA provides a denser functional profile per sequence **(B)** Comparative annotation success rate. The chart illustrates the percentage of transcripts successfully assigned to any functional metadata (“Annotated”) versus those remaining uncharacterized (“Unannotated”). quercusTOA reduced the number of uncharacterized transcripts to 28, compared to 42 in the Trapid pipeline.

The comparative analysis demonstrated that quercusTOA significantly increases the functional recovery rate, as evidenced by assigning metadata to 74.8% (83/111) of the transcripts compared to the 62.2% (69/111) achieved by Trapid. This improvement is further reflected in the reduction of “dark” sequences, where our tool left only 25.2% (28/111) without functional assignment, whereas Trapid left 37.8% (42/111) unannotated. Beyond mere recovery, quercusTOA provided a much higher descriptive granularity; as shown in the distribution analysis, the pipeline achieved a significantly higher mean GO density of approximately 81 terms per sequence, nearly doubling the ∼37 terms per sequence produced by Trapid (Table 5; Figure 3A).

**TABLE 5:** Comparative performance of quercusTOA and Trapid2.0 in the functional annotation of *Quercus pubescens* transcripts.

Metric	quercusTOA	Trapid
Total sequences (N)	111	111
Global annotation (any metadata)	83 (74.8%)	69 (62.2%)
GO term assignment (functional)	65 (58.6%)	63 (56.7%)
Unannotated (no metadata)	28	42
Transcripts w/o GO terms	46	48
Mean GO density	High (∼81)	Mean (∼37)

The table summarizes the annotation metrics for a reference set of 111 simulated “degenerated” transcripts from *Quercus pubescens*. Global Annotation indicates the number and percentage of sequences receiving at least one piece of metadata (e.g., protein signatures, families, or GO terms). GO Term Assignment specifically denotes the subset of sequences with at least one associated Gene Ontology term. Unannotated sequences represent the “dark” transcriptomic fraction with no functional hits in the respective databases. Mean GO density reflects the average number of GO terms assigned per annotated sequence.

These results highlight quercusTOA’s ability to leverage multiple databases evidence to overcome annotation bottlenecks. By doubling the number of functional descriptors per sequence while maintaining a high rate of global assignment (Figure 3B), the platform proves to be a robust resource for the functional characterization of non-model oak species.

## 4 Discussion

The exponential growth of genomic resources for the genus *Quercus* has led to a situation where valuable information remains scattered across fragmented repositories (Zhang et al., 2025). quercusTOA addresses this challenge by providing a centralized and scalable framework that synchronizes functional metadata with genomic positioning, offering a more cohesive biological context for oak research. A key advantage of our integrated architecture is the implementation of genomic lift-over via Liftoff, which represents a significant enhancement over purely protein-centric databases. By maintaining the correspondence between physical genomic coordinates and functional annotations, quercusTOA facilitates comparative analyses across different infrageneric sections. This positional information allows the identification of orthologous relationships and syntenic blocks that are often obscured when analyzing single sequences. Furthermore, our taxonomy-aware approach allows for the characterization of both core and lineage-specific gene clusters at multiple taxonomic levels, providing the necessary resolution to explore the molecular basis of the high adaptive diversity that defines the genus *Quercus*.

The observed U-shaped distribution of orthogroup/cluster sharing across the nine oak genomes reflects a genomic architecture defined by a conserved core alongside a fraction of species-specific or accessory genes. This pattern is consistent with recent evidence for other species complexes, such as *Populus* (Shi et al., 2024), *Oryza* (Guo et al., 2025), and *Solanum* (Benoit et al., 2025). The identification of a significant core genome, representing 43.7% of our oak gene clusters, aligns with the high chromosomal synteny and stable genome size reported for the genus (Plomion et al., 2018; Larson et al., 2025). This structural stability suggests that the core component of quercusTOA captures the shared evolutionary heritage of these species. Furthermore, the significant fraction of lineage-specific or low-frequency clusters (24.8% for oak gene clusters at n = 1 and n = 2) reflects the evolutionary lability observed in specific gene families. As noted by Larson et al. (2025), while the overall oak genome structure is conserved, certain regions, such as those involved in environmental adaptation or disease resistance, exhibit rapid turnover and diversification. Our results, derived from both *de novo* and homology-based strategies, support the overall coherence of quercusTOA and are consistent with established genomic conservation patterns described for the *Quercus* genus (Plomion et al., 2018; Kremer and Hipp, 2020; Larson et al., 2025).

Beyond the database itself, the public availability of our database building scripts represents a significant contribution to the community. By providing the complete automated pipeline in an open format, we empower other researchers to build their own customized databases, allowing them to experiment with different parameters -such as sequence identity thresholds or coverage- and to even incorporate additional genomes as they are released. Moreover, the database generation scripts can be easily adapted to other species complexes at the infrageneric level, provided that enough high-quality genomes from different closely related species are available - i.e., genus *Populus* (Shi et al., 2024). This open-science approach makes it possible that researchers can independently update and adapt the resource to accommodate the continuous influx of new genomic data. This scalability is complemented by the quercusTOA-app, which ensures that these complex resources are easily operable for a broad range of users. By providing a graphical interface that automates local BLAST searches, alignments, and phylogenomic reconstructions, we lower the technical barrier for researchers who may not have extensive command-line expertise, facilitating the biological interpretation of large-scale genomic datasets through an intuitive analytical framework.

Despite these strengths, the utility of the database is inherently influenced by the heterogeneous quality of the original genome assemblies and the diverse pipelines employed for gene inference. Variations in the number of repetitive elements (Rey et al., 2023), high levels of heterozygosity (Kremer et al., 2012) or assembly contiguity (Sork et al., 2022) and annotation depth among the source genomes (Plomion et al., 2018) can impact the success rate of genomic lift-over and the density of functional assignments in oaks. Although most genomes included in quercusTOA share standard protocols for gene modeling, including repeat identification, RNA-seq integration, and homology searches, the specific methods have evolved significantly over time. We chose to rely on the original gene models provided by the authors of each assembly rather than imposing a unified re-annotation protocol. Researchers should, therefore, consider these underlying technical differences when interpreting comparative results across species with divergent assembly levels, such as those at the scaffold versus chromosome level. Future updates of quercusTOA will focus on the continuous integration of new high-quality assemblies and the refinement of functional mapping to maintain the tool as a robust reference. Ultimately, by reconciling fragmented information into a standardized, taxonomy-aware framework, quercusTOA constitutes a valuable resource for future studies in oak evolution and molecular breeding.

## Data Availability

The QUERCUSTOA database is hosted at the UPMDRIVE cloud repository (Universidad Politécnica de Madrid, 2014–2026) and is permanently archived in the ZENODO repository (Mora-Márquez et al., 2026b). The data can be accessed and downloaded via the project website (https://blogs.upm.es/quercustoa/) or using the QUERCUSTOA-APP software available at the GITHUB (https://github.com/GGFHF/quercusTOA-app) and ZENODO repositories (Mora-Márquez et al., 2026a).
