# Supplementary material for: quercusTOA: integrating functional annotations and comparative genomics across oak lineages
Source: Front Bioinform. 2026 Jun 1;6:1821531. doi: 10.3389/fbinf.2026.1821531 (PMC13265531; doi:10.3389/fbinf.2026.1821531)
Supplement: Supplementary file 1 [file DataSheet1.pdf]

frontiers

QUERCUSTOA: Integrating functional annotations and comparative genomics across oak lineages

Supplementary Material

SUPPLEMENTARY FILE 1: Description of the SQLite databases of the QUERCUSTOA database. It is composed of three `.db` files, each with several tables:

SQLite DATABASE 1: `sequences.db`

Table 1.1: `species_protein_seqs`

Column	Type	Index	Comment
protein_id	TEXT	1	protein identification
species_id	TEXT		species identification
seq	TEXT		sequence of the protein

Table 1.2: `species_gene_seqs`

Column	Type	Index	Comment
gene_id	TEXT	1	gene identification
species_id	TEXT		species identification
seq	TEXT		sequence of the protein

SQLite DATABASE 2: `comparative-genomics.db`

Table 2.1: `liftoff_gff_cds_data`

Column	Type	Index	Comment
reference_species_id	TEXT	1,2	identification of the reference species
target_species_id	TEXT	1	identification of the target species
reference_protein_id	TEXT	2	protein identification of the reference species
target_seq_id	TEXT	1	sequence identification of the reference species
target_start	INT		start position in the reference species genome
target_end	INT		end position in the reference species genome
target_strand	TEXT		strand in the reference species genome

Table 2.2: `liftoff_gff_gene_data`

Column	Type	Index	Comment
reference_species_id	TEXT	1	identification of the reference species
target_species_id	TEXT	1	identification of the target species
reference_gene_id	TEXT	2	gene identification of the reference species
target_seq_id	TEXT	1	sequence identification of the reference species
target_start	INT		start position in the reference species genome
target_end	INT		end position in the reference species genome
target_strand	TEXT		strand in the reference species genome
reference_protein_id_list	TEXT		protein identification list of the reference species

Table 2.3: `liftoff_homologous_proteins`

Column	Type	Index	Comment
reference_species_id	TEXT	1	identification of the reference species
target_species_id	TEXT	1	identification of the target species
reference_protein_id	TEXT	1,2	protein identification of the reference species
target_protein_id	TEXT		protein identification of the target species

Table 2.4: `liftoff_unmapped_genes`

Column	Type	Index	Comment
reference_species_id	TEXT	1	identification of the reference species
target_species_id	TEXT	1	identification of the target species
reference_gene_id	TEXT	1,2	gene identification of the reference species

Table 2.5: `mmseqs2_concatenated_cds_clusers`

Column	Type	Index	Comment
species_id	TEXT	1,2,3	species identification
cluster_id	TEXT	1	identification of concatenated CDS cluster
seq_id	TEXT	2	sequence identification
gene_id	TEXT		gene identification
protein_id	TEXT	3	protein identification

SQLite DATABASE 3: `functional-annotation.db`

Table 3.1: `mmseq2_protein_clusters`

Column	Type	Index	Comment
cluster_id	TEXT	1	identification of protein cluster
seq_id	TEXT	2	NCBI protein sequence identification
description	TEXT		description from the NCBI protein sequence
species	TEXT		species from the NCBI protein sequence

Table 3.2: `interproscan_annotations`

Column	Type	Index	Comment
cluster_id	TEXT	1	cluster identification
interpro_goterms	TEXT		concatenated list of GO terms from InterPro
panther_goterms	TEXT		concatenated list of GO terms from Panther
x_goterms	TEXT		concatenated list of GO terms from other sources
metacyc_pathways	TEXT		concatenated list of pathway identifications from MetaCyc
reactome_pathways	TEXT		concatenated list of pathway identifications from Reactome
x_pathways	TEXT		concatenated list of pathway identifications from other sources

Table 3.3: `tair10_info`

Column	Type	Index	Comment
tair10_peptide_id	TEXT	1	*A. thaliana* peptide identification
description	TEXT		Description of the *A. thalina* peptide

Table 3.4: `tair10_orthologs`

Column	Type	Index	Comment
cluster_id	TEXT	1	cluster identification
ortholog_seq_id	TEXT		ortholog sequence identification of *A. thalina*

Table 3.5: `go_ontology`

Column	Type	Index	Comment
go_id	TEXT	1	GO term identification
go_name	TEXT		GO term description
namespace	TEXT		Molecular function, biological process or cellular component

Table 3.6: `emapper_annotations`

Column	Type	Index	Comment
cluster_id	TEXT	1	cluster identification
ortholog_seq_id	TEXT		ortholog sequence identification from eggNOG
ortholog_species	TEXT		species from eggNOG
eggnog_ogs	TEXT		OGs (Orthologous Groups) of proteins from eggNOG
cog_category	TEXT		COG (Cluster of Orthologous Genes) from eggNOG
description	TEXT		description from eggNOG
goterms	TEXT		concatenated list of GO terms from eggNOG
ec	TEXT		concatenated list of EC (Enzyme Commission) numbers
kegg_kos	TEXT		concatenated list of KO from KEGG
kegg_pathways	TEXT		concatenated list of pathway identifications from KEGG
kegg_modules	TEXT		concatenated list of module identifications from KEGG
kegg_reactions	TEXT		concatenated list of chemical reactions identifications from KEGG
kegg_rclasses	TEXT		concatenated list of reactions classification identifications from KEGG
brite	TEXT		functional hierarchy of OGs assigned to the sequence
kegg_tc	TEXT		T cell receptor (TCR) signaling pathway
cazy	TEXT		concatenated list of Carbohydrate-Active Enzymes (CAZymes)
pfams	TEXT		concatenated list of protein families from Pfam
